# Temporal changes in the fecal bacterial community in Holstein dairy calves from birth through the transition to a solid diet

**DOI:** 10.1371/journal.pone.0238882

**Published:** 2020-09-08

**Authors:** Meagan L. Hennessy, Nagaraju Indugu, Bonnie Vecchiarelli, Joseph Bender, Christa Pappalardo, Miranda Leibstein, John Toth, Ananya Katepalli, Satvik Garapati, Dipti Pitta

**Affiliations:** 1 Department of Clinical Studies, School of Veterinary Medicine, University of Pennsylvania, Kennett Square, PA, United States of America; 2 Oceanside High School, Oceanside, NY, United States of America; 3 Northwest High School, Germantown, MD, United States of America; 4 Drexel University, Philadelphia, PA, United States of America; University of Maine, UNITED STATES

## Abstract

The development of a robust microbiome is critical to the health of dairy calves, but relatively little is known about the progression of the microbiome through the weaning transition. In this study, fecal samples were obtained from ten female Holstein calves at 6 timepoints between 2–13 weeks of age. Calves were fed acidified milk until weaning at 8 weeks old and had access to starter grain throughout the study. Fecal samples were extracted for genomic DNA, PCR-amplified for the V1-V2 region of the 16S rRNA bacterial gene, sequenced on the Illumina MiSeq platform, and analyzed using the QIIME2 pipeline. Bacterial richness, estimated by number of observed species, and bacterial diversity, estimated by Shannon diversity index, both differed significantly between timepoints and both increased over time (*P* <0.05), with the largest increases occurring during weaning. Weighted and unweighted UniFrac analysis showed significant differences (*P* <0.05) between bacterial communities across timepoints; betadisper analysis revealed that the microbiomes of individual calves became more similar with time. Throughout the study, Firmicutes was the dominant phylum, followed by Bacteroidetes. Thirteen bacterial genera were found to be significantly influenced by time, including *Faecalibacterium*, *Clostridium*, unclassified *S24-7*, *Collinsella*, *Sharpea*, and *Treponema*. Unclassified *Ruminococcaceae* was the most prevalent genus at timepoints 1, 3, 5, and 6, but different amplicon sequence variants were detected at each timepoint suggesting the presence of different species of *Ruminococcaceae* at different times. *Bacteroides* was the most prevalent genus at timepoint 2, and *Prevotella* was most prevalent at timepoint 4. Our results indicate that there is considerable variation in the calf microbiome pre-weaning, but the microbial community stabilizes and becomes similar to the adult microbiome at weaning. Further studies to describe the phylogeny and functionality of core microbiota through the weaning transition are needed to improve health and reduce diarrhea in the neonatal period.

## Introduction

The development of a robust microbiome is essential to the health of growing calves, but relatively little is known about the calf microbiome through the weaning transition, when calves transition from milk to solid feed as their main feed source. Pre-weaned calves are vulnerable to gastrointestinal illness; according to the 2007 National Animal Health Monitoring System (NAHMS) for the US dairy industry, 57% of mortality in pre-weaned calves was caused by diarrhea, predominantly in calves less than one month old [1]. Diarrhea in calves can be caused by enteric pathogens including viruses, bacteria, and protozoa [2, 3], but the cause is often multifactorial and co-infection with multiple pathogens is common [4, 5]. A functioning microbiome has been shown to be key to the establishment of a functional immune system. As an example, the interaction between beneficial bacteria such as *Lactobacillus* and *Bifidobacterium* and the gut mucosa has been shown to be necessary for the proper formation of tight junctions in the gastrointestinal tract (GIT) in the neonatal period; alterations to the microbiome at this stage can lead to increased gut permeability and an increased risk of infection [6, 7].

The microbiome has been shown to be very dynamic during the first twelve weeks of a calf’s life, as their digestive system develops and their feed sources change [8, 9]. The GIT of the calf is rapidly populated by microorganisms from the birth canal, external environment, colostrum, and feed in the immediate neonatal period [10]. Neonatal calves are considered functional monogastrics, with the majority of their digestion taking place in the abomasum, which constitutes almost 50% of the entire stomach at birth; conversely, as calves develop, begin eating solid fermentable feed, and become true ruminants, the reticulorumen becomes dominant [10, 11]. Studies have shown that mammals, including calves, tend to follow a pattern of colonization during the immediate neonatal period, with the first colonizers being facultative anaerobes such as *Escherichia coli* and *Lactobacillus*. These facultative anaerobes help to establish the reduced environment necessary for anaerobes such as *Bifidobacterium* [6, 8, 12]. These studies indicate the presence of a core microbiome in neonatal calves; however, comprehensive studies examining the microorganisms present during the development of healthy calves beyond the immediate neonatal period and through the weaning transition are lacking, leaving the question of what constitutes a healthy versus dysbiotic microbiome during this stage of development.

The purpose of this study was to gain a better understanding of the changes in the fecal microbiota of the calf from shortly after birth through the post-weaning period. To this end, we conducted a cohort study with a group of 10 female Holstein dairy calves housed together and analyzed fecal samples at 6 timepoints between 16–20 days of age and 86–90 days of age. We hypothesized that there would be significant flux in the fecal microbiome during this period and that there would be significant changes as the calves transitioned to a solid diet.

## Materials/Methods

### Study design and sample collection

All animal studies were approved by the University of Pennsylvania Institutional Animal Care and Use Committee (IUCAC protocol #80614).

A cohort of 10 female Holstein calves (C1 –C10) from one dairy farm in Southeastern Pennsylvania were enrolled in the study at 16–20 days old, and sampled every other week until reaching 86–90 days of age for a total of 6 sampling timepoints. Sampling took place between June and August of 2019. Calves had been moved into individual hutches immediately after birth and given 1 gallon of acidified colostrum by orogastric tube within 1 hour of birth. Between 7 and 10 days of age, calves were moved into a group pen (pen dimensions: approximately 43x12 feet) that was bedded with sawdust and straw. Calves were offered acidified milk (amounts are given in S1A Table); once moved into the group pen, the acidified milk was fed via a communal trough. Calves had free choice access to a calf starter grain containing monensin from approximately 2 weeks of age (see S1A Table for grain intake), and free choice hay (approximately 3 flakes per calf per day) began to be offered approximately 3 days after weaning. Weaning occurred at approximately 8 weeks of age, the day before sampling timepoint (TP) 4. The components of the starter grain were as follows: monensin 72 g/ton, crude protein min. 18%, crude fat min. 4.25%, crude fiber max. 8% (further diet information given in S1B Table).

Fecal samples were obtained via rectal stimulation and stored immediately on ice for transport to the laboratory, where they were frozen at -20°C until extraction for DNA (approximately 1.5–2 months after collection). A sample was not obtained for calf 10 at sampling TP1; all other samples were obtained as planned for a total of 59 samples.

### DNA extraction, amplification, and sequencing

Genomic DNA was extracted from approximately 250 mg of each fecal sample using the repeated bead beating and column (RBB + C) method followed by extraction with a commercial kit (QIAmp Fast DNA Stool Mini Kit; Qiagen Sciences, Germantown, MD) based on the procedure described in Yu and Morrison [13]. Extracted samples were stored at -20°C for approximately 1–2 months before sequencing. For each extracted genomic DNA sample, the V1-V2 region of the bacterial 16S rRNA gene was PCR-amplified in triplicate using the bacterial-specific primers F27 (5′-AGAGTTTGATCCTGGCTCAG-3′) and R338 (5′-TGCTGCCTCCCGTAGGAGT-3′) barcoded with a unique 12-base error-correcting Golay code for multiplexing as described in Song et al. [14]. Polymerase chain reaction was performed in triplicate using the Accuprime Taq DNA Polymerase System (Invitrogen; Carlsbad, CA). The thermal cycling conditions involved an initial denaturing step at 95° C for 5 min followed by 20 cycles (denaturing at 95° C for 30 sec, annealing at 56° C for 30 sec, extension at 72° C for 90 sec) and a final extension step at 72° C for 8 min. The amplicons from each DNA sample were combined and each library was added to a pool in equimolar concentration. Two DNA extraction blanks and two PCR amplification blanks were included in the final pool. The final pool was bead purified using Beckman Coulter Agencourt AMPure XP Beads (Beckman Coulter; Brea, CA). Sequencing was performed at the PennCHOP Microbiome Core, University of Pennsylvania, using the Illumina MiSeq platform.

### Bioinformatics and statistical analysis

The bacterial amplicon sequences were processed through the QIIME2 (2018.4) pipeline [15]. The raw sequences were de-multiplexed and assigned to amplicon sequence variants (ASV) using the DADA2 plugin [16] according to the following parameters: the input sequences reads were truncated at the 3 frame end of the sequence at 230 nucleotides and default settings were used for the remaining options of this plugin. Multiple sequence alignment was carried out with MAFFT [17] and sequences were filtered to remove highly variable positions using default settings. FastTree 2 [18] was used to construct and root a phylogenetic tree using default settings. Taxonomic classification was conducted using a pre-trained Naive Bayes classifier trained on the Greengenes (v13.8) database for the 16S rRNA region spanning the V1-V2 region [19].

Alpha diversity was assessed via ‘observed_otus’ and ‘shannon’ and beta diversity was measured using weighted and unweighted UniFrac distances. The measured alpha diversity matrices were compared between timepoints using the Wilcoxon/Kruskal-Wallis Rank Sum test. A nonparametric permutational multivariate ANOVA test [20], implemented in the vegan package for R, was used for beta diversity matrices. The analysis of multivariate homogeneity of group dispersions (variances) was calculated using the betadisper function available in R vegan packages.

The raw read counts from the 16S rRNA ASV abundance table were collapsed at taxonomic rank and compositionally normalized (relative abundance) such that each sample summed to 1. Analysis of composition of microbiomes (ANCOM) tests were run in QIIME2 at genus level taxonomic classification to determine which genera were differentially abundant between timepoints. Spearman correlations were calculated to test the correlation between bacterial genera. Genera were considered present if their average percentage across all timepoints was at least 0.01% and the correlation were considered significant if *P* ≤ 0.01.

### Sequence availability

The fecal microbiome sequences have been deposited in the NCBI database under BioProject accession number PRJNA612881 and under the Sequence Read Archive (SRA) accession IDs of SRX7930363 –SRX7930421.

## Results

### Sequencing details

A total of 3,051,582 raw sequencing reads were generated from a total of 59 samples, with a mean (± SD) of 51,722 (± 14,826) reads per sample. Less than 100 reads per sample were observed in the blank samples (two DNA blanks and two PCR blanks) and these samples were dropped from the analysis. This produced a total of 7,954 ASV. Representative sequences from these ASV were assigned to 16 bacterial phyla. A total of 158 genera were identified in this study.

### Within sample variation (alpha diversity)

**Fig 1:**
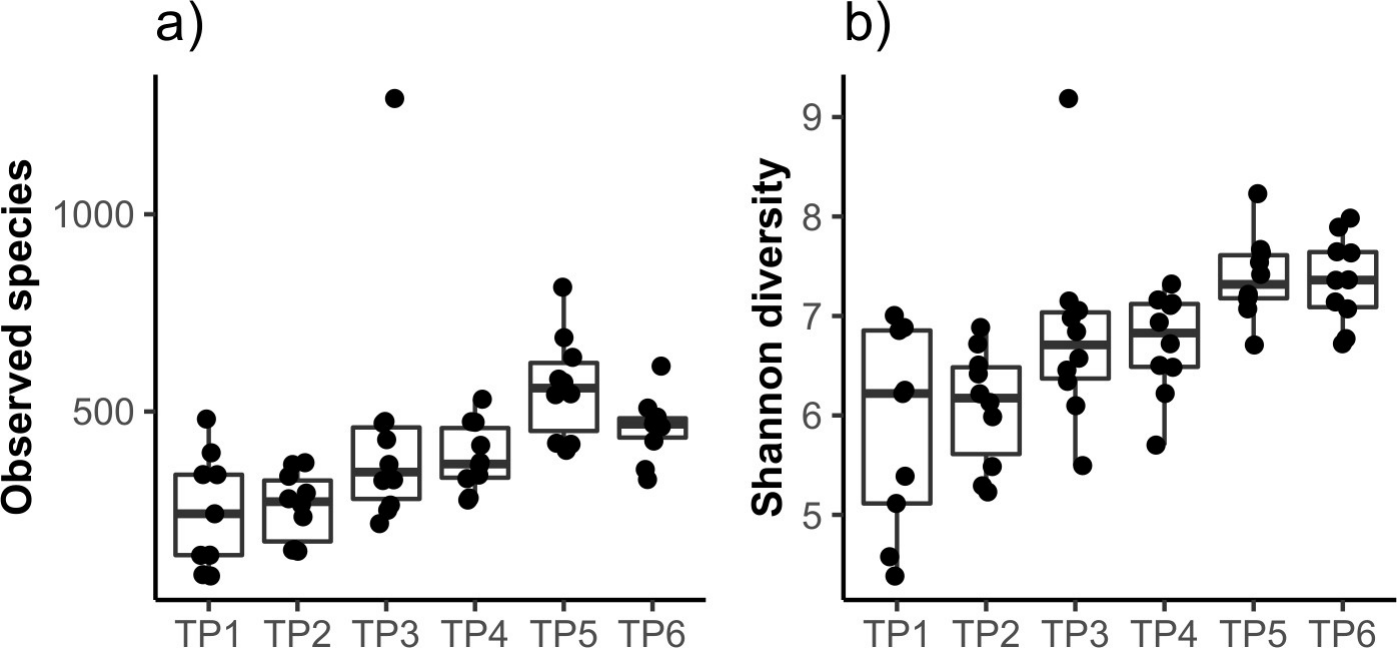
(a,b) Within-sample diversity (alpha diversity). Measurement of within-sample variation (alpha diversity) of the fecal bacterial communities of the group of 10 calves at each of 6 sampling timepoints. (a) number of observed species at each timepoint and (b) Shannon diversity at each timepoint. TP = timepoint.

To analyze alpha diversity or within-sample variation, individual results from the 10 calves were grouped by timepoint for analysis of the number of bacterial species (observed species; estimated by species richness) and the distribution of bacterial species within each community (estimated by Shannon diversity index) (Fig 1A and 1B; S2 Table). Both species richness and Shannon diversity showed an overall increase with calf age (*P* <0.05 for both; Kruskal-Wallis test), and this increase was particularly pronounced in species richness. Both measures of alpha diversity were similar between TP1 and TP2, between TP3 and TP4, and between TP5 and TP6 (*P* >0.05 for these comparisons; Wilcoxon test), indicating that there was less fluctuation in individual samples between these timepoints. Conversely, there was a significant difference (*P* <0.05; Wilcoxon test) between TP2 and TP3 and between TP4 and TP5 in Shannon diversity, and a significant difference between TP4 and TP5 in observed species, indicating more substantial changes in individual microbiota between these timepoints.

### Between bacterial community comparison (beta diversity)

**Fig 2:**
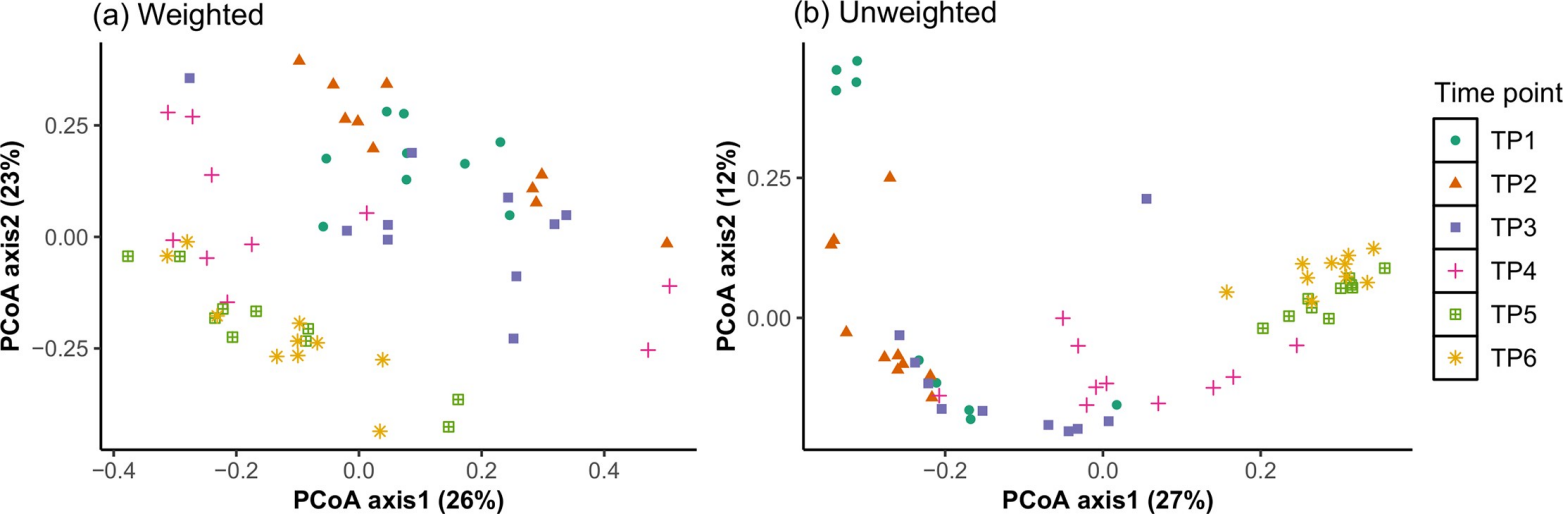
(a,b) Between bacterial community comparisons (beta diversity). Comparison of bacterial community composition between timepoints using principal coordinate analysis (PCoA). (a) Weighted UniFrac distances based on relative abundance of bacterial ASV and (b) Unweighted UniFrac distances based on presence/absence information of bacterial ASV. TP = timepoint.

The weighted and unweighted UniFrac distances were used to derive principal coordinates (PCoA) (Fig 2A and 2B; S3 Table). In both weighted and unweighted measures, the timepoints differed from each other (*P* <0.05; PERMANOVA test) and showed clear clustering by age, particularly in the unweighted analysis. Based on PCoA, bacterial communities appeared to become more similar between individual calves at later timepoints, with communities at TP6 being the most similar between individual calves, indicating a convergence of the microbial populations from individual calves over time. In unweighted UniFrac, betadisper analysis supported this (F = 2.315; *P* = 0.044; S4 Table) and revealed that individual variation in the community structure was significantly greater in TP1 as compared to TP2, TP5, and TP6 (pairwise comparisons; *P* <0.05). However, in the weighted UniFrac, betadisper analysis found no difference (F = 1.044; *P* = 0.412) in bacterial community dispersion in individuals between timepoints.

### Phylogenetic composition of fecal bacterial communities

**Fig 3:**
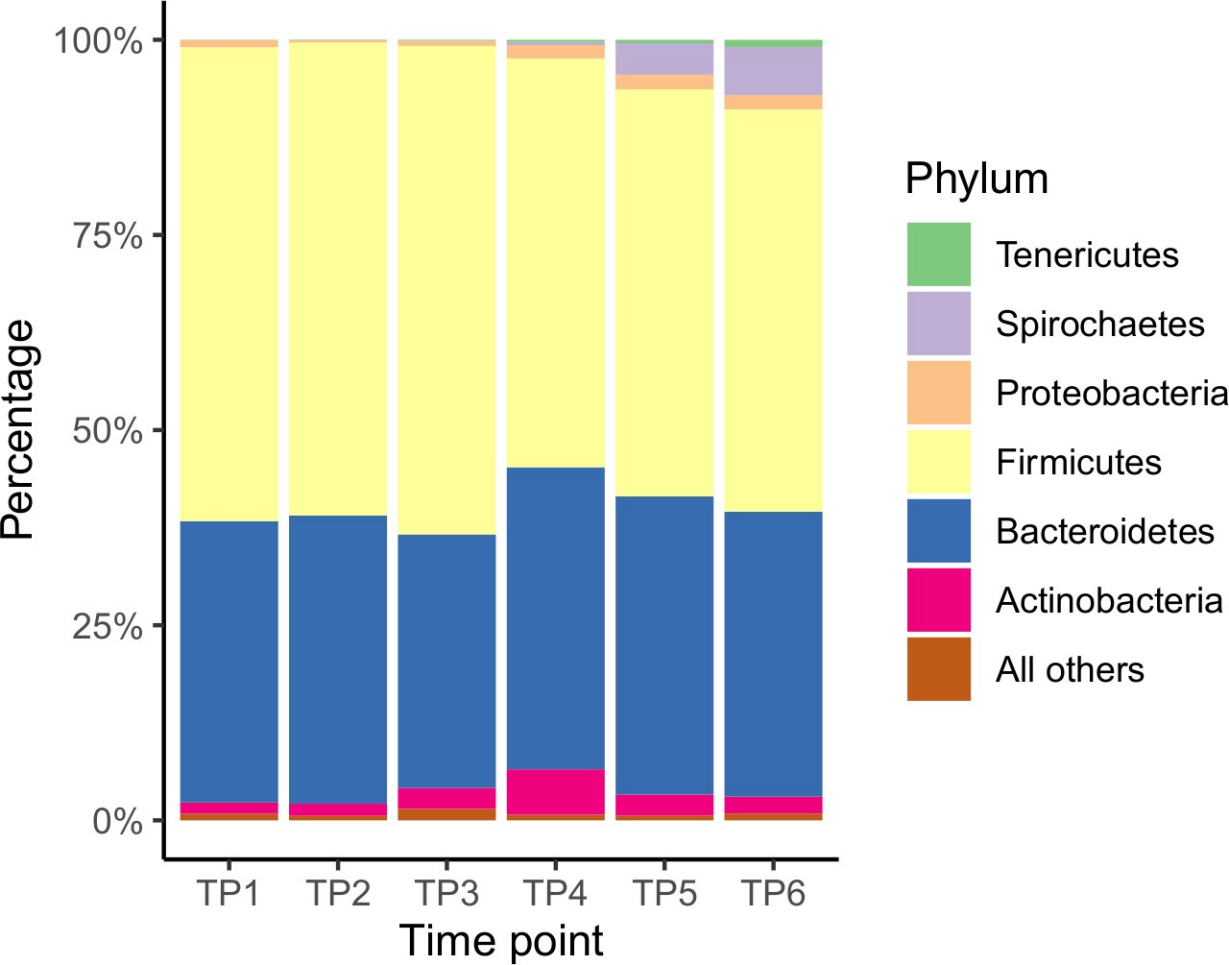
Relative abundance of individual bacterial phyla. Mean value of relative abundance (given as percentage) of the top 6 phyla at each timepoint. TP = timepoint.

When the phylum-level results for each of the 10 individual calves were averaged together by timepoint, Firmicutes was the dominant phylum at each of the 6 timepoints (ranging from 51.5% relative abundance at TP6 to 62.6% at TP3), followed by Bacteroidetes (ranging from 32.5% at TP3 to 38.7% at TP4; Fig 3; S5 Table). For TP1 through 4, Actinobacteria, Proteobacteria, and Cyanobacteria made up the third through fifth most prevalent phyla, although their order differed. Cyanobacteria was present at low but stable abundance throughout the study, ranging from 0.4% at TP5 to 1.2% at TP3. Actinobacteria made up <2.8% of phyla at TP1-3, then increased to 5.8% at TP4 before declining again to <2.8% at TP5-6. For TP5 and 6, Spirochaetes, which made up <1% of TP1-4, became the third most prevalent phyla (average of 4.0% of TP5 and 6.2% of TP6). Although Firmicutes was the dominant phyla throughout the course of the study, it showed a non-linear decline with time (from 60.7% at TP1 to 51.5% at TP6).

At the genus level, there were clear shifts demonstrated by timepoint (S1 Fig; S6 Table). At TP1, when calves were between 16 and 20 days old, the most prevalent genera were unclassified *Ruminococcaceae* (Firmicutes; 29.0%), followed by *Bacteroides* (Bacteroidetes; 28.3%), unclassified *Lachnospiraceae* (Firmicutes; 6.6%), *Parabacteroides* (Bacteroidetes; 3.9%), *Blautia* (Firmicutes; 3.5%), and *Faecalibacterium* (Firmicutes; 2.6%). At TP2, the 6 most abundant genera remained the same, although their relative abundance varied; the most prevalent genus was *Bacteroides* (24.3%), followed by *Faecalibacterium* (16.5%), unclassified *Ruminococcaceae* (14.6%), *Parabacteroides* (9.6%), *Blautia* (6.7%), and unclassified *Lachnospiraceae* (5.1%). These timepoints were the least diverse, with the 6 most abundant genera listed here making up 73.8% (TP1) and 76.9% (TP2) of the total populations, indicating the presence of a core microbiome at this phase during the calves’ development.

At TP3, when the calves were between 44 and 48 days old, the most dominant genus was again unclassified *Ruminococcaceae* (22.2%), followed by *Bacteroides* (16.9%), unclassified *Lachnospiraceae* (8.4%), *Parabacteroides* (7.7%), unclassified *Clostridiales* (Firmicutes; 6.2%), and *Faecalibacterium* (5.5%). At TP4, when the calves had been weaned for <24 hours, *Prevotella* (Bacteroidetes) became the most dominant genus (16.5%), followed by *Bacteroides* (11.5%), unclassified *Ruminococcaceae* (10.3%), unclassified *Lachnospiraceae* (7.7%), *Blautia* (7.0%), and unclassified *S24-7* (Bacteroidetes; 6.3%). These timepoints showed increased diversity compared to TP1 and TP2, with the 6 most abundant genera making up 67.0% (TP3) and 57.3% (TP4) of the total populations.

At TP5, when the calves were between 72 and 76 days old and had been weaned for 15 days, unclassified *Ruminococcaceae* was again the most prevalent (18.8%), followed by unclassified *S24-7* (10.6%), *Prevotella* (10.4%), unclassified *Lachnospiraceae* (10.3%), unclassified *Clostridiales* (7.4%), and *Bacteroides* (6.2%). At TP6, unclassified *Ruminococcaceae* remained the most prevalent, although it had declined in abundance to 15.6%; it was followed by unclassified *S24-7* (13.3%), unclassified *Lachnospiraceae* (10.9%), *Prevotella* (9.9%), *Bacteroides* (6.5%), and *Treponema* (Spirochaetes; 6.2%). The 6 most abundant genera at these timepoints made up 63.8% (TP5) and 62.4% (TP6) of the total populations.

Of the less prevalent genera, unclassified *Coriobacteriaceae* (Actinobacteria) was present at low but relatively stable levels throughout the study, ranging between 0.2% (TP2) and 5.6% (TP4). *CF231* (Bacteroidetes) made up 6.1% of genera at TP5 but was <1.8% at all other timepoints. Lastly, *Sutterella*, the most prevalent genus from the Proteobacteria phylum, was ≤1.0% at all timepoints.

**Fig 4:**
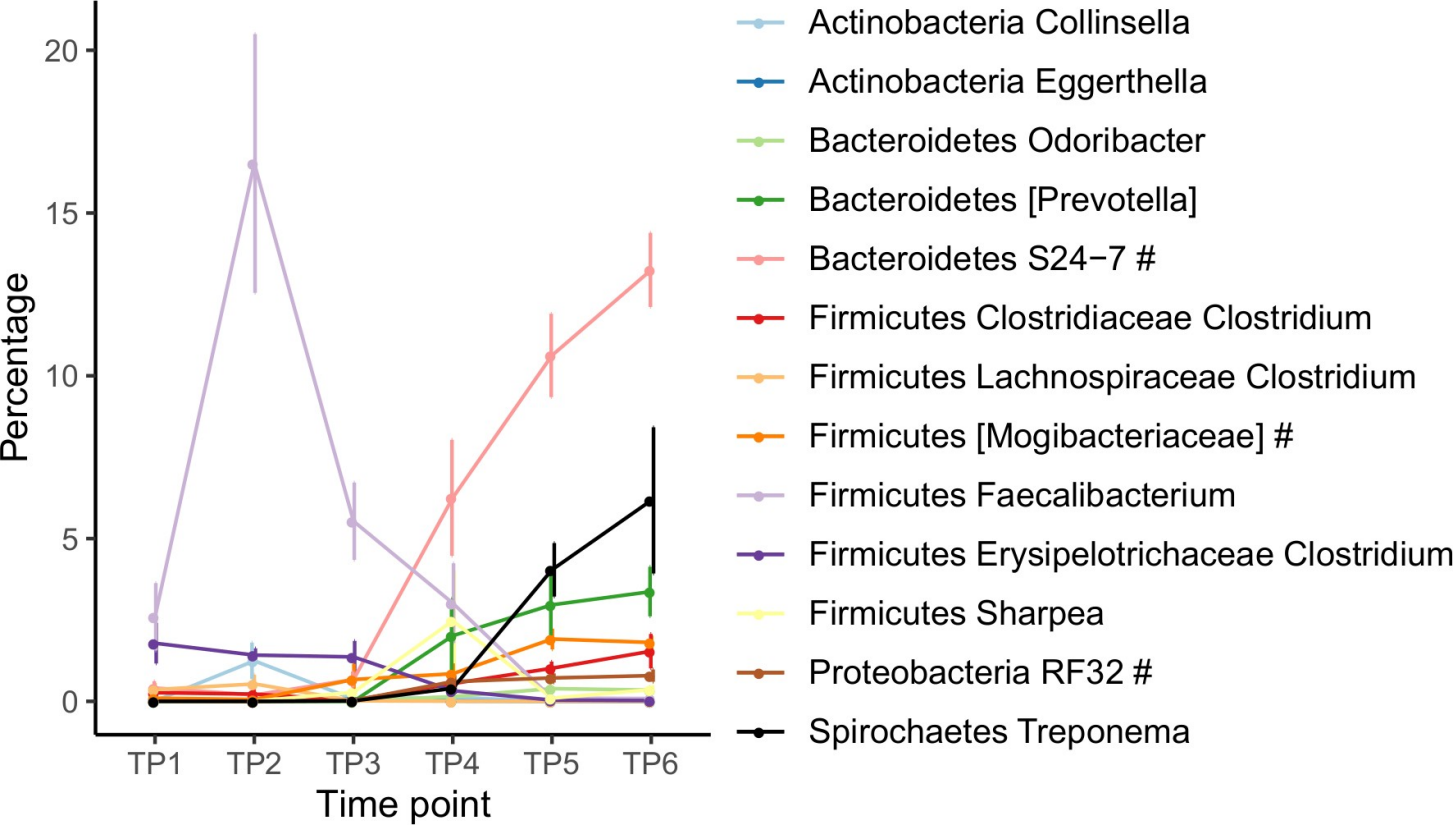
Changes in relative abundance of significant bacterial genera. Changes in the relative abundance of genera that differed between timepoints as analyzed by ANCOM test. TP = timepoint. The symbol # beside genus name indicates unclassified genus from the family, order, or class given.

A total of 13 genera were found to be significantly influenced by time using the ANCOM test (Fig 4). As discussed above, *Faecalibacterium* made up 16.5% of the total genera at TP2 and 5.5% of total genera at TP3 but represented <3.0% of total genera at all other timepoints. Unclassified *S24-7* showed a large increase in relative abundance after TP3, making up 6.3, 10.6, and 13.3% of total genera at TP4, 5, and 6, respectively. Similarly, *Treponema* increased significantly with time; it made up <0.4% of total genera at TP1-4, then increased to 4.0 and 6.2% at TP5 and 6. In addition, less prevalent genera such as *Collinsella*, *Eggerthella*, *Odoribacter*, unclassified *Mogibacteriaceae*, *Sharpea*, and *RF32* were also found to be significantly influenced by time, although they did not make up >5% of total genera at any timepoint.

**Fig 5:**
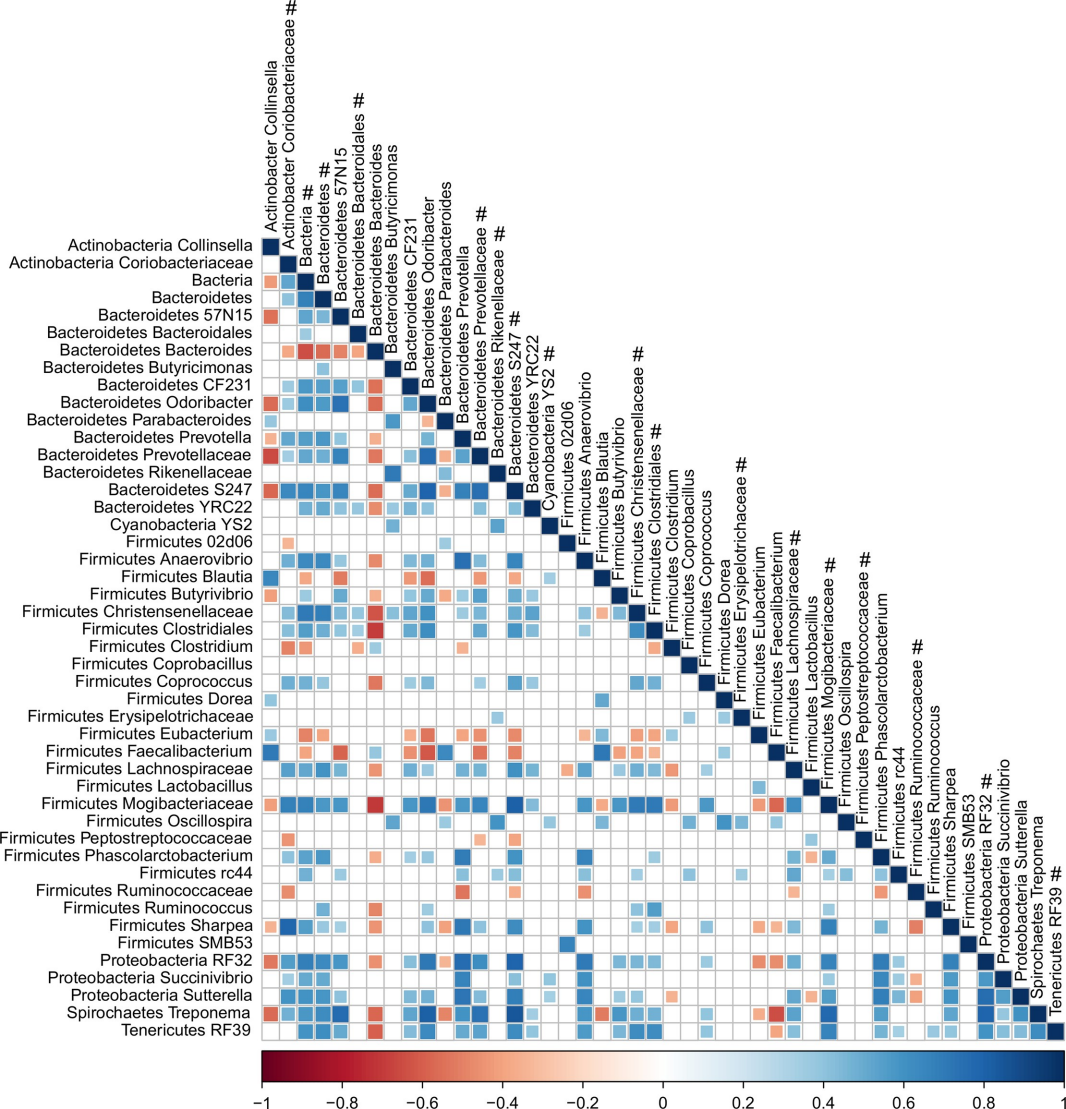
Correlations between individual bacterial genera. Interactions among bacterial genera as revealed by correlation plot. Only statistically significant correlations (P ≤ 0.01) are shown. The symbol # beside genus name indicates unclassified genus from the family, order, class, phylum, or kingdom given.

To determine associations between bacterial genera, a correlation analysis was performed (Fig 5). Unclassified *Ruminococcaceae* was significantly (*P* <0.01) negatively correlated with unclassified *Coriobacteriaceae*, *Prevotella*, unclassified *S24-7*, unclassified *Lachnospiraceae*, and *Sharpea*. *Faecalibacterium* was significantly positively correlated with *Collinsella*, *Bacteroides*, *Parabacteroides*, and *Blautia*, and significantly negatively correlated with unclassified *Mogibacteriaceae*, *Sharpea*, *Treponema*, and unclassified *S24-7*. *Bacteroides* showed significant positive correlations with *Clostridium* and significant negative correlations with unclassified *S24-7*, unclassified *Lachnospiraceae*, unclassified *Mogibacteriaceae*, and *RF32*. *Treponema* was significantly positively correlated with *Sharpea*, unclassified *Coriobacteriaceae*, *Prevotella*, unclassified *S24-7*, unclassified *Lachnospiraceae*, and unclassified *Mogibacteriaceae*, and significantly negatively correlated with *Collinsella*, *Bacteroides*, *Parabacteroides*, and *Blautia*.

**Fig 6:**
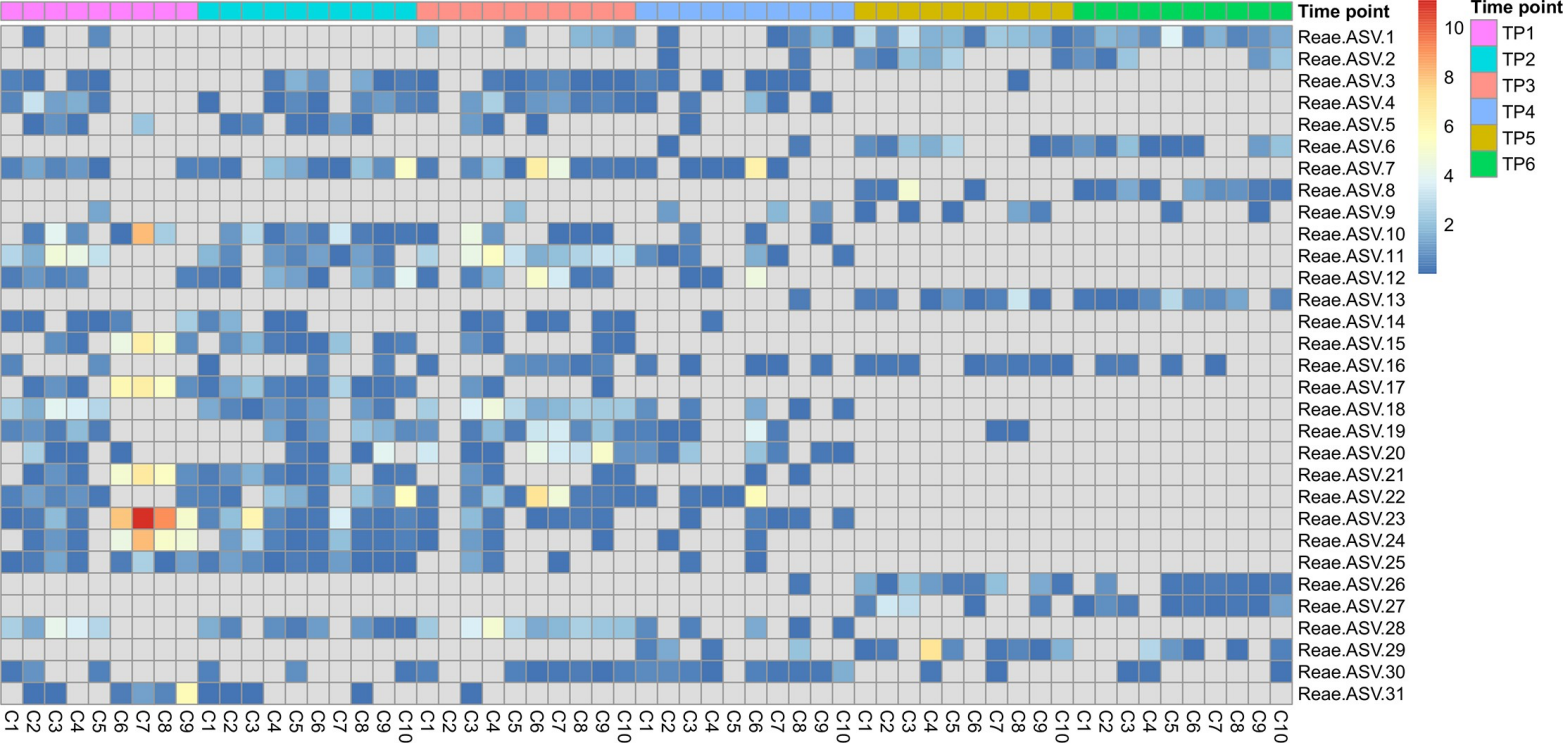
Relative abundance of amplicon sequence variants of unclassified genera of Ruminococcaceae. Heat map showing the prevalence of amplicon sequence variants (ASV) of unclassified genera of Ruminococcaceae in each individual calf sample (C1-C10, bottom axis) at each timepoint (TP1-6, top axis). Relative abundance (%) of each ASV is indicated by color (color scale given in upper right-hand corner). Grey squares indicate absence of an ASV in that sample.

Lastly, because unclassified *Ruminococcaceae* were found to be prevalent at all timepoints throughout the study, we analyzed the ASV of unclassified *Ruminococcaceae* at the genus level at each timepoint. Of the 31 ASV analyzed representing unclassified *Ruminococcaceae* species there were notable trends between timepoints, with the makeup of ASV varying greatly (Fig 6). As an example, the 5 most abundant ASV at TP1 (ASV 15, 17, 21, 23, and 24) were all reduced by more than half by TP2 and were not present at all in TP5 or TP6. Similarly, the 5 most abundant ASV at TP5 were not present at TP1 and 2 and were present in low amounts at TP3 and 4. TP5 and TP6, the post-weaning timepoints, were similar in their makeup of ASV.

## Discussion

The development of a robust microbiome has been shown to be crucial to the health and development of dairy calves. It is necessary for a functioning immune system and may provide some measure of protection against the gastrointestinal disorders that are common in pre-weaned calves [6]. In adult dairy cows, the intestinal microbiome has been linked not only to health outcomes but also to measures of productivity, such as milk production and feed efficiency [21, 22]. The microbiome of calves has been shown to be highly variable, and a more detailed understanding of the progression of the calf microbiome from the neonatal period through weaning will lend insight into what constitutes a healthy versus dysbiotic microbiome at these crucial stages of growth and development. To that end, this study sought to determine changes in the fecal microbiome in a cohort of 10 calves sampled 6 times from an average of 18 days of age through an average of 88 days of age.

In the immediate neonatal period, calves have been shown to follow a classic mammalian pattern of colonization, with facultative anaerobes such as *Escherichia coli* and *Lactobacillus* establishing the reduced environment necessary for beneficial anaerobes such as *Bifidobacterium* [6, 8, 12]. These organisms are transferred to the calf through intake of colostrum, which contains organisms such as *Staphylococcus*, *Ruminococcaceae*, *Clostridiales*, *Escherichia coli*, and *Prevotella*, as well as through transfer from the environment and the dam [23–25]. In a study that examined calves sampled at one timepoint between the ages of 1 and 30 days, *Lactobacillus*, *Bifidobacterium*, and *Escherichia-Shigella* were found to be the dominant genera in both healthy and diarrheic calves [26]. Interestingly, *Enterobacteriaceae*, the genus that contains the *Escherichia* species, was found in only a few of the samples from this study, with its highest prevalence being 1.7% in one calf at TP1, and *Bifidobacterium* was not found in any samples. *Lactobacillus* made up 26.8% of the genera found in the sample from one calf at TP1 but was <1% of all other samples. It is likely that because the calves in our study were not sampled for the first time until 16–20 days of age, these initial colonizers were not identified; additionally, sequencing for the V1-V2 region of the 16S bacterial gene has been shown to lead to poor recovery of *Bifidobacterium*, which could have contributed to the fact that it was not found in any samples in this study [27].

At TP1 in the present study, the calves were 16–20 days old, had been living in the group pen for 1–2 weeks, and had been offered starter grain for approximately 2–7 days. This timepoint was found to have the highest degree of variation between calves (based on beta diversity and betadisper analysis), and also to have the lowest number of observed species and lowest Shannon diversity. This timepoint was dominated by unclassified *Ruminococcaceae* (29.0%) and *Bacteroides* (28.3%), with the top six genera representing 73.8% of total identified genera. This agrees with findings in both neonatal calves and human infants where initial microbial colonization of the GIT is limited to a relatively small number of species at first but progressively becomes more diverse with age [8, 12]. A recent study found that unassigned OTU from the *Ruminococcaceae* family were the most abundant genus in both the vaginal and fecal microbiome of cows who had just given birth [24], and they are also present in colostrum [23]. The 5 most abundant ASV at TP1 were present at TP2 at less than half the abundance, had largely disappeared by TP3 and TP4, and were not present at all at TP5 and TP6; therefore, we speculate that the distinct profile of unclassified *Ruminococcaceae* that was present at this timepoint may have been transferred from the calves by their dams.

At TP2, calves were 30–34 days old and were consuming the highest amount of acidified milk at any point in the study. The acidification of waste milk for use as a calf feed is fairly common in the US, and has been shown to reduce the growth of pathogens in the milk and allow it to be stored without refrigeration [28]. Acidified milk has also been shown to be effective in reduction of the incidence of calf diarrhea as well as in growth promotion [29–31], but there are few studies examining its effect on the microbiome. A recent study comparing calves fed acidified waste milk with calves fed either pasteurized waste milk, untreated bulk milk, or untreated waste milk found that the calves fed acidified or pasteurized waste milk had higher proportions of *Faecalibacterium*, *Bacteroides*, and *Prevotella* in their feces at 21 days old than the other two groups, which had higher proportions of *Fusobacterium* [28]. This agrees with the finding of our study, where at TP2, *Bacteroides*, *Faecalibacterium*, *Blautia*, and *Parabacteroides* were among the most prevalent genera. *Faecalibacterium prausnitzii*, the only known species of *Faecalibacterium*, has been found to correlate with increased weight gain and a reduction in the prevalence of diarrhea in calves [32], and has been successfully used as an oral probiotic supplement in calves to prevent the incidence of and mortality from diarrhea [33]. *Faecalibacterium* is able to metabolize acetate to butyrate, a volatile fatty acid that is not only the major fuel source of colonocytes but also stimulates their growth and differentiation [34]. We found that *Faecalibacterium* was significantly (*P* <0.01) positively correlated with acetate-producing bacteria such as *Blautia* and *Bacteroides* as well as with succinate producer *Parabacteroides;* these organisms formed the core microbiome supported by acidified milk at TP2 and declined thereafter. A study of 3-week-old dairy calves found that *Faecalibacterium* made up 11% of the genera in the fecal microbiome [9], and it represented 12.1% of the genera in the colon of 3-week-old steer calves in another study [8], further confirming that this genus is a major role player at this stage in development and may contribute significantly to promoting gastrointestinal growth and health.

The relative abundance of unclassified *Ruminococcaceae* at TP2 was approximately half what it was at TP1 (14.6% versus 29.0%), and the analysis of ASV showed that different species were present at this timepoint. This indicates that although the species found in TP1 may have been transferred from the calves’ dams, these species were beginning to be lost by TP2 and were being replaced by those that were able to digest a primarily milk-based diet. Further studies are needed to better understand which species of *Ruminococcaceae* are present and active at different stages of development.

At TP3, calves were 44–48 days old. They were still receiving acidified milk but the amount per day was declining, and they were beginning to increase their consumption of starter grain to compensate. This timepoint resembled TP2 except for the significant decline of *Faecalibacterium;* the dominant genera were unclassified *Ruminococcaceae*, *Bacteroides*, unclassified *Lachnospiraceae*, *Parabacteroides*, and unclassified *Clostridiales*. The individual microbiomes of the calves were becoming more diverse at this point (Shannon diversity; *P* <0.05 for comparison between TP2 and TP3), with the 6 most abundant genera representing 67.0% of total identified genera.

At TP4, the calves were 58–62 days old and had been weaned for approximately 24 hours. Hay had not yet been added to their diets, making starter grain with monensin their only feed source. Monensin, an ionophore antibiotic, has been reported to improve feed utilization and dairy cow production by increasing digestibility of neutral detergent fiber and non-fibrous carbohydrate [35], possibly due to increased ruminal feed retention time [36], and increasing ruminal production of propionate [37]. In addition, by inhibiting Gram-positive pathogenic bacteria, it has been shown to decrease rates of diarrhea [35, 38]. To date, there have been relatively few studies exploring the changes in the microbiome caused by monensin or the effect of introducing monensin to calves. A study done in multiparous dairy cows found that cows treated with monensin had an increase in abundance of succinate-producing *Prevotella* and butyrate-producing *S24-7* in their rumen as compared to control cows [37], which agrees with our findings for TP4-TP6. Another recent study found that treatment of multiparous lactating cows with monensin led to a dose-dependent decrease in relative abundance of Gram-positive Firmicutes accompanied by an increase in relative abundance of Gram-negative Bacteroidetes and Proteobacteria in the rumen [39], which also agrees with our results for the post-weaning timepoints in this study. However, the considerable differences between rumen versus fecal microbiota as well as the difference in the microbiome in adult dairy cows versus calves make these results difficult to compare. More studies are needed on how monensin could alter the microbiome in calves when it is fed during the weaning transition.

TP4 also saw an increase in *Coriobacteriaceae* (Actinobacteria), from <2.4% in TP1-3 to 5.6%. This family was the major representative from the phylum Actinobacteria and was largely responsible for the increase seen in the phylum at this time point. It has been shown to be responsive to changes in diet [40] and transfaunation [41], and has also been shown to be more prevalent in pasture-fed dairy cows [42]. It is likely that the increase in this family was a result of the increase in solid feed intake at this time point.

Between TP4 (24 hours post-weaning) and TP5 (15 days post-weaning), there were significant differences in both alpha and beta diversity measures (*P* <0.05 for all); however, there was no significant difference in alpha or beta diversity measurements between these TP5 and TP6, and betadisper analysis indicated that the there was little variation between individual calves at these timepoints. These results indicate that by TP5 (72–26 days old) and TP6 (86–90 days old), the microbiota of the calves had largely stabilized. Unclassified *Ruminococcaceae*, which had showed a decline at TP4, again became the most dominant genus at TP5 and TP6 (18.8% and 15.6%, respectively); as discussed above, however, the 5 most prevalent ASV at these timepoints were more than twice as abundant at these timepoints than at earlier timepoints, indicating the presence of different species within unclassified *Ruminococcaceae*. This increase in unclassified *Ruminococcaceae* was accompanied by increases in unclassified *S24-7* (10.6 and 13.3% at TP5 and TP6, respectively) and *Treponema* (4.0 and 6.2% at TP5 and TP6, respectively). Members of the family *S24-7* are butyrate producers and have been shown to positively correlate with milk yield in dairy cows [43, 44]. The increased presence of the Spirochaete *Treponema* is likely due to the increase of fiber in the diet at these timepoints, at which the calves were subsisting solely on grain and hay; *Treponema* is a fibrolytic bacteria that is able to digest hemicellulose, and it has been shown to increase in abundance in the rumen of sheep fed high-fiber diets [45].

Our results indicate that there is considerable variation in the calf microbiome pre-weaning, but the microbial community stabilizes and becomes similar to the adult microbiome at weaning. The weaning transition is accompanied by large shifts in the microbiome as the calf begins to subsist solely on fermentable solid feed instead of milk, and fermentation in the rumen rather than the intestine becomes the dominant source of energy for the calf. The strengths of this study include the fact that it is one of the few studies to analyze the microbiome of calves as they move through the weaning transition. Although the relatively small sample size and the fact that calves were sampled from only one farm are limitations, this study nonetheless provides valuable information on this crucial period in calf development as well as gives insight into possible effects of acidified milk and monensin on the fecal microbiota in the developing calf. Future research should include a larger number of calves and should begin sampling at an earlier age to clarify differences in the individual microbiota of calves before being separated from their dams and before being moved into group pens. Additionally, future work should include comparisons with sick calves, with calves from other farms, and with calves fed different diets.

## Supporting information

S1 Table Information on calf diets. **(a)** Volume of acidified milk and amount of starter grain fed to calves throughout course of study; **(b)** composition of calf starter grain.
(DOCX)

S2 Table Statistical significance for measures of alpha diversity. Statistical significance (Wilcoxon Rank Sum Test) for measures of alpha diversity. P values are given for between-timepoint comparisons for both observed species and Shannon diversity. Significant P values (<0.05) are in bold. TP = timepoint.
(DOCX)

S3 Table Statistical significance for measures of beta diversity. Statistical significance (PERMANOVA test) for measures of beta diversity. P values are given for between-timepoint comparisons for both weighted and unweighted UniFrac analysis. Significant P values (<0.05) are in bold. TP = timepoint.
(DOCX)

S4 Table Betadisper analysis. Permutation test for homogeneity of multivariate dispersions (betadisper analysis). Pairwise comparisons were performed between each timepoint in both weighted (a) and unweighted (b) UniFrac analysis. Timepoint comparisons with significant differences in beta diversity (P <0.05) are in bold. Overall P values for beta dispersion: weighted = 0.412; unweighted = 0.044.
(DOCX)

S5 Table Relative abundance of individual bacterial phyla. Relative abundances (given by percentage) of individual phyla present at each timepoint. TP = timepoint.
(DOCX)

S6 Table Relative abundance of individual bacterial genera. Relative abundances (given by percentage) of individual genera present at each timepoint. TP = timepoint.
(DOCX)

S1 Fig Relative abundance of individual bacterial genera. Heat map showing the relative abundance of genera in each of the 10 calves (C1-C10, bottom axis) sampled at each of the 6 study timepoints (TP, top axis). Relative abundance (%) of each genus is indicated by color (color scale given in upper right-hand corner). The symbol # beside genus name indicates unclassified genus from the family, order, class, phylum, or kingdom given. Grey squares indicate absence of a genus in that sample.
(TIFF)
