# Supplementary material for: Temporal changes in the fecal bacterial community in Holstein dairy calves from birth through the transition to a solid diet
Source: PLoS One. 2020 Sep 8;15(9):e0238882. doi: 10.1371/journal.pone.0238882 (PMC7478546; doi:10.1371/journal.pone.0238882)
Supplement: S1 Table — (a) Volume of acidified milk and amount of starter grain fed to calves throughout course of study; (b) composition of calf starter grain. (DOCX) [file pone.0238882.s001.docx]

**Supplemental table 1 a, b**

**(a) Amount of acidified milk and starter grain b) Composition of starter grain**

**given per calf per week**

| Monensin | 72 g/ton |
| --- | --- |
| Crude protein | Minimum 18.0% |
| Crude fat | Minimum 4.25% |
| Crude fiber | Maximum 8.0% |
| Ingredients | Grain products, plant protein products, processed grain, byproducts, molasses, vegetable oil, calcium carbonate, blood meal, salt, vitamin E supplement, mineral oil, premium yeast fraction, sodium selenite, vitamin A acetate/D3 supplement, copper sulfate, magnesium oxide, magnesium sulfate, potassium sulfate, zinc sulfated, ethylenediamine, dihydroidide, cobalt carbonate |

| **Calf age (weeks)** | **Volume of acidified milk given per calf 2x/day** | **Amount of starter grain given per calf 1x/day** |
| --- | --- | --- |
| 1 | 2 quarts | 0 lbs  2 |
| 2 | 3 quarts | 2 lbs |
| 3 | 4 quarts | 2.5 lbs |
| 4 | 5 quarts | 3 lbs |
| 5 | 4 quarts | 3.5 lbs |
| 6 | 3 quarts | 4 lbs |
| 7 | 2 quarts | 4.5 lbs |
| 8 | Weaning | 5 lbs |
