# Supplementary material for: Temporal changes in the fecal bacterial community in Holstein dairy calves from birth through the transition to a solid diet
Source: PLoS One. 2020 Sep 8;15(9):e0238882. doi: 10.1371/journal.pone.0238882 (PMC7478546; doi:10.1371/journal.pone.0238882)
Supplement: S2 Table — Statistical significance (Wilcoxon Rank Sum Test) for measures of alpha diversity. P values are given for between-timepoint comparisons for both observed species and Shannon diversity. Significant P values (<0.05) are in bold. TP = timepoint. (DOCX) [file pone.0238882.s002.docx]

| **Time point** | **Observed Species** | **Shannon Diversity** | **Time point** | **Observed Species** | **Shannon Diversity** |
| --- | --- | --- | --- | --- | --- |
| TP1 vs TP2 | 0.713062 | 0.842105 | TP2 vs TP6 | **0.000765** | **4.33E-05** |
| TP1 vs TP3 | 0.130573 | 0.078893 | TP3 vs TP4 | 0.578742 | 0.684211 |
| TP1 vs TP4 | 0.078916 | **0.034987** | TP3 vs TP5 | **0.018543** | **0.006841** |
| TP1 vs TP5 | **0.000695** | **0.000152** | TP3 vs TP6 | 0.10398 | **0.023231** |
| TP1 vs TP6 | **0.006165** | **0.00065** | TP4 vs TP5 | **0.001505** | **0.003886** |
| TP2 vs TP3 | 0.081984 | **0.043257** | TP4 vs TP6 | 0.130281 | **0.011496** |
| TP2 vs TP4 | **0.008931** | **0.01469** | TP5 vs TP6 | 0.10398 | 0.911797 |
| TP2 vs TP5 | **1.08E-05** | **4.33E-05** |  |  |  |

**Supplemental table 2**
