# Supplementary material for: Temporal changes in the fecal bacterial community in Holstein dairy calves from birth through the transition to a solid diet
Source: PLoS One. 2020 Sep 8;15(9):e0238882. doi: 10.1371/journal.pone.0238882 (PMC7478546; doi:10.1371/journal.pone.0238882)
Supplement: S3 Table — Statistical significance (PERMANOVA test) for measures of beta diversity. P values are given for between-timepoint comparisons for both weighted and unweighted UniFrac analysis. Significant P values (<0.05) are in bold. TP = timepoint. (DOCX) [file pone.0238882.s003.docx]

| **Time point** | **Weighted UniFrac** | **Unweighted UniFrac** | **Time point** | **Weighted UniFrac** | **Unweighted UniFrac** |
| --- | --- | --- | --- | --- | --- |
| TP1 vs TP2 | **0.039** | **0.037** | TP2 vs TP6 | **0.001** | **0.001** |
| TP1 vs TP3 | **0.038** | 0.056 | TP3 vs TP4 | **0.029** | **0.004** |
| TP1 vs TP4 | **0.001** | **0.002** | TP3 vs TP5 | **0.001** | **0.001** |
| TP1 vs TP5 | **0.002** | **0.001** | TP3 vs TP6 | **0.001** | **0.001** |
| TP1 vs TP6 | **0.001** | **0.001** | TP4 vs TP5 | **0.005** | **0.001** |
| TP2 vs TP3 | **0.029** | **0.001** | TP4 vs TP6 | **0.006** | **0.001** |
| TP2 vs TP4 | **0.006** | **0.001** | TP5 vs TP6 | 0.143 | **0.01** |
| TP2 vs TP5 | **0.001** | **0.001** |  |  |  |

**Supplemental table 3**
