# Supplementary material for: Temporal changes in the fecal bacterial community in Holstein dairy calves from birth through the transition to a solid diet
Source: PLoS One. 2020 Sep 8;15(9):e0238882. doi: 10.1371/journal.pone.0238882 (PMC7478546; doi:10.1371/journal.pone.0238882)
Supplement: S4 Table — Permutation test for homogeneity of multivariate dispersions (betadisper analysis). Pairwise comparisons were performed between each timepoint in both weighted (a) and unweighted (b) UniFrac analysis. Timepoint comparisons with significant differences in beta diversity (P <0.05) are in bold. Overall P values for beta dispersion: weighted = 0.412; unweighted = 0.044. (DOCX) [file pone.0238882.s004.docx]

**Supplemental table 4 a, b**

**a) Weighted analysis b) Unweighted analysis**

|  | **TP1** | **TP2** | **TP3** | **TP4** | **TP5** | **TP6** |
| --- | --- | --- | --- | --- | --- | --- |
| **TP1** |  | **0.008** | 0.430 | 0.052 | 0.001 | 0.001 |
| **TP2** | **0.010** |  | 0.362 | 0.214 | 0.996 | 0.963 |
| **TP3** | 0.433 | 0.323 |  | 0.873 | 0.271 | 0.304 |
| **TP4** | 0.060 | 0.201 | 0.838 |  | 0.053 | 0.056 |
| **TP5** | **0.000** | 0.996 | 0.266 | 0.057 |  | 0.914 |
| **TP6** | **0.000** | 0.950 | 0.282 | 0.070 | 0.908 |  |

|  | **TP1** | **TP2** | **TP3** | **TP4** | **TP5** | **TP6** |
| --- | --- | --- | --- | --- | --- | --- |
| **TP1** |  | 0.955 | 0.869 | 0.224 | 0.792 | 0.729 |
| **TP2** | 0.945 |  | 0.901 | 0.217 | 0.731 | 0.657 |
| **TP3** | 0.876 | 0.912 |  | 0.287 | 0.708 | 0.651 |
| **TP4** | 0.212 | 0.197 | 0.287 |  | 0.125 | 0.099 |
| **TP5** | 0.788 | 0.702 | 0.690 | 0.114 |  | 0.918 |
| **TP6** | 0.739 | 0.657 | 0.652 | 0.113 | 0.914 |  |
