# Supplementary material for: Temporal changes in the fecal bacterial community in Holstein dairy calves from birth through the transition to a solid diet
Source: PLoS One. 2020 Sep 8;15(9):e0238882. doi: 10.1371/journal.pone.0238882 (PMC7478546; doi:10.1371/journal.pone.0238882)
Supplement: S5 Table — Relative abundances (given by percentage) of individual phyla present at each timepoint. TP = timepoint. (DOCX) [file pone.0238882.s005.docx]

**Supplemental table 5**

| **Phylum** | **TP1** | **TP2** | **TP3** | **TP4** | **TP5** | **TP6** |
| --- | --- | --- | --- | --- | --- | --- |
| Actinobacteria | 1.445816 | 1.551308 | 2.663977 | 5.818807 | 2.695451 | 2.171419 |
| Bacteroidetes | 36.05028 | 36.93195 | 32.48308 | 38.68861 | 38.19336 | 36.49323 |
| Chloroflexi | 0 | 0 | 0 | 0 | 0 | 0 |
| Cyanobacteria | 0.620406 | 0.531945 | 1.180689 | 0.604529 | 0.360985 | 0.612719 |
| Elusimicrobia | 0.074555 | 0 | 0.032317 | 0.036034 | 0.066373 | 0.034915 |
| Fibrobacteres | 0 | 0 | 0.021924 | 0 | 0 | 0 |
| Firmicutes | 60.71369 | 60.59587 | 62.58949 | 52.39078 | 52.16692 | 51.54682 |
| Fusobacteria | 0.001208 | 0.00182 | 0 | 0.000541 | 0.002247 | 0 |
| Other | 0.122649 | 0.001793 | 0.140699 | 0.077233 | 0.157722 | 0.217074 |
| Proteobacteria | 0.908207 | 0.269667 | 0.63758 | 1.704093 | 1.854554 | 1.823709 |
| Spirochaetes | 0 | 0 | 0.016385 | 0.40034 | 4.04143 | 6.173556 |
| SR1 | 0 | 0 | 0.002308 | 0 | 0 | 0 |
| Synergistetes | 0 | 0 | 0 | 0 | 0 | 0 |
| Tenericutes | 0.034491 | 0.069382 | 0.120527 | 0.27492 | 0.437761 | 0.914601 |
| TM7 | 0 | 0 | 0.034386 | 0 | 0 | 0 |
| Verrucomicrobia | 0.028704 | 0.046263 | 0.072259 | 0.004108 | 0.0232 | 0.011955 |
| WPS2 | 0 | 0 | 0.004385 | 0 | 0 | 0 |
