# Supplementary material for: Temporal changes in the fecal bacterial community in Holstein dairy calves from birth through the transition to a solid diet
Source: PLoS One. 2020 Sep 8;15(9):e0238882. doi: 10.1371/journal.pone.0238882 (PMC7478546; doi:10.1371/journal.pone.0238882)
Supplement: S6 Table — Relative abundances (given by percentage) of individual genera present at each timepoint. TP = timepoint. (DOCX) [file pone.0238882.s006.docx]

**Supplemental table 6**

| **Genus** | **TP1** | **TP2** | **TP3** | **TP4** | **TP5** | **TP6** |
| --- | --- | --- | --- | --- | --- | --- |
| *Actinobacteria Actinomyces* | 0 | 0.00051019 | 0.0022876 | 0.00050334 | 0 | 0 |
| *Actinobacteria Actinomycetales* | 0.0011817 | 0.00145376 | 0.00075447 | 0.000755 | 0 | 0 |
| *Actinobacteria Adlercreutzia* | 0 | 0.00275585 | 0.00589361 | 0.02439195 | 0.01905605 | 0.01160716 |
| *Actinobacteria Arthrobacter* | 0 | 0 | 0 | 0 | 0 | 0 |
| *Actinobacteria Atopobium* | 0 | 0 | 0.03369412 | 0 | 0 | 0 |
| *Actinobacteria Bifidobacterium* | 0.00451392 | 0 | 0 | 0 | 0 | 0 |
| *Actinobacteria Collinsella* | 0.01188499 | 1.24507062 | 0.07001657 | 0.12163202 | 0.00338995 | 0 |
| *Actinobacteria Coriobacteriaceae* | 1.34220957 | 0.24458961 | 2.4461615 | 5.64555802 | 2.6554132 | 2.14108536 |
| *Actinobacteria Corynebacterium* | 0.00462897 | 0.00204076 | 0.00436354 | 0.00536026 | 0.00608784 | 0.00139587 |
| *Actinobacteria Curtobacterium* | 0 | 0 | 0 | 0 | 0.00193094 | 0 |
| *Actinobacteria Dietzia* | 0.00098468 | 0.00195775 | 0.00502877 | 0.00411327 | 0 | 0.00179582 |
| *Actinobacteria Eggerthella* | 0.07082089 | 0.03265544 | 0.07459286 | 0.0106117 | 0.00295157 | 0 |
| *Actinobacteria Geodermatophilaceae* | 0 | 0 | 0 | 0.0005108 | 0 | 0 |
| *Actinobacteria Glycomyces* | 0 | 0 | 0.00046156 | 0 | 0 | 0 |
| *Actinobacteria Intrasporangiaceae* | 0 | 0 | 0 | 0.000755 | 0 | 0 |
| *Actinobacteria Kocuria* | 0 | 0 | 0 | 0 | 0 | 0 |
| *Actinobacteria Leucobacter* | 0 | 0 | 0 | 0.00100667 | 0 | 0 |
| *Actinobacteria Microbacteriaceae* | 0 | 0 | 0.00050014 | 0.00159543 | 0 | 0.00566922 |
| *Actinobacteria Microbacterium* | 0.00322076 | 0.00186306 | 0.00302196 | 0.00151 | 0.00472295 | 0.00852605 |
| *Actinobacteria Microbispora* | 0 | 0.00034147 | 0 | 0 | 0 | 0 |
| *Actinobacteria Micrococcaceae* | 0 | 0 | 0 | 0 | 0 | 0 |
| *Actinobacteria Mycetocola* | 0 | 0 | 0.00092313 | 0 | 0 | 0 |
| *Actinobacteria Mycobacterium* | 0 | 0 | 0 | 0 | 0 | 0.00067486 |
| *Actinobacteria Nocardiopsaceae* | 0 | 0 | 0 | 0 | 0 | 0.0006645 |
| *Actinobacteria Propionibacteriaceae* | 0.00039396 | 0 | 0 | 0 | 0.00034786 | 0 |
| *Actinobacteria Pseudoclavibacter* | 0 | 0 | 0 | 0 | 0 | 0 |
| *Actinobacteria Rhodococcus* | 0 | 0 | 0 | 0 | 0 | 0 |
| *Actinobacteria Saccharopolyspora* | 0 | 0.00076529 | 0 | 0 | 0.00093353 | 0 |
| *Actinobacteria Slackia* | 0.00479484 | 0.01212091 | 0.01476462 | 0 | 0 | 0 |
| *Actinobacteria Streptomyces* | 0 | 0.00348898 | 0.00105119 | 0.00050334 | 0 | 0 |
| *Actinobacteria Trueperella* | 0.00118161 | 0.00169451 | 0.00046156 | 0 | 0.00061749 | 0 |
| *Bacteria* | 0.12264947 | 0.00179343 | 0.1406994 | 0.07723336 | 0.15772168 | 0.21707397 |
| *Bacteroidetes* | 0.12842085 | 0.10185881 | 0.16311015 | 0.30567946 | 0.91281655 | 0.3514238 |
| *Bacteroidetes 57N15* | 0 | 0 | 0 | 0.55424496 | 0.98257128 | 1.12718912 |
| *Bacteroidetes Bacteroidales* | 0.00086375 | 0 | 0.04709682 | 1.92223217 | 0.2374312 | 1.51730877 |
| *Bacteroidetes Bacteroides* | 28.2755281 | 24.346466 | 16.8939831 | 11.4866097 | 6.22095081 | 6.45639623 |
| *Bacteroidetes Barnesiellaceae* | 0 | 0 | 0 | 0 | 0.0041417 | 0 |
| *Bacteroidetes BF311* | 0 | 0 | 0.00115391 | 0 | 0 | 0 |
| *Bacteroidetes BS11* | 0 | 0 | 0 | 0 | 0 | 0 |
| *Bacteroidetes Butyricimonas* | 0.71001675 | 0.67330129 | 0.71564868 | 0.10988398 | 0.28381697 | 0.14538465 |
| *Bacteroidetes CF231* | 1.47318164 | 0 | 1.26766052 | 0.23824474 | 6.092884 | 1.75884761 |
| *Bacteroidetes Chitinophagaceae* | 0 | 0 | 0 | 0 | 0 | 0 |
| *Bacteroidetes Odoribacter* | 0 | 0 | 0 | 0.14823762 | 0.39134005 | 0.34634094 |
| *Bacteroidetes p253418B5* | 0 | 0 | 0.00069235 | 0 | 0 | 0 |
| *Bacteroidetes Paludibacter* | 0.00430126 | 0 | 0.0067348 | 0 | 0.03509025 | 0 |
| *Bacteroidetes Parabacteroides* | 3.85889869 | 9.61919252 | 7.73405835 | 0.96186078 | 1.15556441 | 1.11970519 |
| *Bacteroidetes Paraprevotellaceae* | 0 | 0 | 0.02169348 | 0 | 0 | 0 |
| *Bacteroidetes Porphyromonadaceae* | 0.00310647 | 0 | 0.00238315 | 0.00325388 | 0 | 0 |
| *Bacteroidetes Porphyromonas* | 0 | 0 | 0 | 0 | 0 | 0 |
| *Bacteroidetes Prevotella* | 0.80032993 | 1.77604506 | 4.61073268 | 16.480679 | 10.4368147 | 9.92756029 |
| *Bacteroidetes Prevotellaceae* | 0.02894955 | 0 | 0.03307964 | 0.10171401 | 0.33554584 | 0.20944328 |
| *Bacteroidetes RF16* | 0 | 0 | 0.00346173 | 0 | 0 | 0 |
| *Bacteroidetes Rikenellaceae* | 0.21832734 | 0.13883473 | 0.2004408 | 0.11994766 | 0.17839566 | 0.12941708 |
| *Bacteroidetes S247* | 0.40860365 | 0.19593728 | 0.66996961 | 6.25331333 | 10.626675 | 13.2532697 |
| *Bacteroidetes Sphingobacterium* | 0 | 0 | 0 | 0 | 0 | 0 |
| *Bacteroidetes YRC22* | 0.13974817 | 0.08031722 | 0.11117813 | 0.00271157 | 0.29932133 | 0.15094131 |
| *Chloroflexi JG30KFCM45* | 0 | 0 | 0 | 0 | 0 | 0 |
| *Cyanobacteria Chloroplast* | 0 | 0.01692487 | 0 | 0 | 0 | 0 |
| *Cyanobacteria Streptophyta* | 0 | 0 | 0.01223143 | 0 | 0.00067405 | 0 |
| *Cyanobacteria YS2* | 0.62040564 | 0.51502022 | 1.16845741 | 0.60452869 | 0.3603112 | 0.61271941 |
| *Elusimicrobia Elusimicrobiaceae* | 0 | 0 | 0.00069235 | 0 | 0.00734598 | 0.01826089 |
| *Elusimicrobia Elusimicrobium* | 0.07455518 | 0 | 0.03162426 | 0.03603391 | 0.05902686 | 0.01665416 |
| *Fibrobacteres Fibrobacter* | 0 | 0 | 0.02192426 | 0 | 0 | 0 |
| *Firmicutes* | 0.06264183 | 0.00048414 | 0.07363038 | 0.01745777 | 0.03664875 | 0.05833585 |
| *Firmicutes 02d06* | 0 | 1.02556961 | 0.00101556 | 0 | 0 | 0 |
| *Firmicutes Acidaminococcus* | 0 | 0 | 0.00923127 | 0.02136789 | 0.00329862 | 0.00897166 |
| *Firmicutes Aerococcaceae* | 0 | 0 | 0 | 0 | 0.00469614 | 0 |
| *Firmicutes Aerococcus* | 0.0011948 | 0 | 0 | 0 | 0.00104359 | 0 |
| *Firmicutes Alicyclobacillus* | 0 | 0 | 0 | 0.0005108 | 0 | 0 |
| *Firmicutes Ammoniphilus* | 0 | 0 | 0.00064082 | 0 | 0 | 0 |
| *Firmicutes Anaerofustis* | 0 | 0.00105937 | 0.00660763 | 0.00249488 | 0.00055223 | 0 |
| *Firmicutes Anaerorhabdus* | 0.00577797 | 0 | 0 | 0 | 0 | 0 |
| *Firmicutes Anaerostipes* | 0.05042033 | 0 | 0.03815559 | 0.04683173 | 0.0810999 | 0.05311962 |
| *Firmicutes Anaerotruncus* | 0 | 0 | 0 | 0 | 0 | 0 |
| *Firmicutes Anaerovibrio* | 0.19057905 | 0.26482705 | 0.43218098 | 3.1228918 | 0.53016248 | 3.40177277 |
| *Firmicutes Anaerovorax* | 0 | 0 | 0 | 0 | 0.02467769 | 0.0432532 |
| *Firmicutes Asteroleplasma* | 0 | 0 | 0.00069235 | 0 | 0 | 0 |
| *Firmicutes Bacillaceae* | 0.00038263 | 0 | 0 | 0 | 0 | 0 |
| *Firmicutes Bacillales* | 0 | 0 | 0 | 0 | 0 | 0 |
| *Firmicutes Bacilli* | 0.00038584 | 0 | 0 | 0 | 0 | 0 |
| *Firmicutes Bacillus* | 0 | 0 | 0.00094132 | 0 | 0.00101698 | 0.00067486 |
| *Firmicutes Blautia* | 3.53454822 | 6.70775282 | 4.87335098 | 7.03535287 | 0.86293694 | 0.68160451 |
| *Firmicutes Bulleidia* | 0 | 0 | 0.11469848 | 0 | 0.02689885 | 0.04334223 |
| *Firmicutes Butyrivibrio* | 0 | 0 | 1.41192218 | 0.00222226 | 0.09611322 | 0.03685573 |
| *Firmicutes Caldicoprobacter* | 0 | 0.0028716 | 0 | 0 | 0.00154652 | 0 |
| *Firmicutes Candidatus Arthromitus* | 0 | 0 | 0 | 0.00444478 | 0 | 0 |
| *Firmicutes Catonella* | 0 | 0 | 0.00069235 | 0 | 0 | 0 |
| *Firmicutes Christensenella* | 0.00116563 | 0.01397541 | 0.00154308 | 0.00634134 | 0.02773225 | 0.01818808 |
| *Firmicutes Christensenellaceae* | 0.02798605 | 0.00866884 | 0.17883121 | 0.03904696 | 0.19983767 | 0.14075321 |
| *Firmicutes Clostridia* | 0.11493733 | 0.03517941 | 0.13077228 | 0.00484672 | 0 | 0.008726 |
| *Firmicutes Clostridiaceae* | 0.02660296 | 0.05547361 | 0.00075306 | 0 | 0.01043961 | 0.01351009 |
| *Firmicutes Clostridiales* | 2.49661203 | 2.34688508 | 6.22690313 | 5.51174098 | 7.40724382 | 5.89570506 |
| *Firmicutes Clostridium* | 2.42602122 | 2.17996358 | 1.42477382 | 0.87696112 | 1.05696062 | 1.56828165 |
| *Firmicutes Coprobacillus* | 0.14573653 | 0.34854587 | 0.14129336 | 0.05410966 | 0.02441966 | 0.07272815 |
| *Firmicutes Coprococcus* | 0.06307817 | 0.19731328 | 0.44860216 | 0.32663064 | 0.43032375 | 0.57425474 |
| *Firmicutes Dehalobacteriaceae* | 0 | 0 | 0 | 0 | 0.07507529 | 0.0485589 |
| *Firmicutes Dehalobacterium* | 0 | 0 | 0.0007008 | 0.0018981 | 0.00561018 | 0.00408932 |
| *Firmicutes Dialister* | 0.0004047 | 0.00126401 | 0.0641573 | 0.02880247 | 0.00875009 | 0.00676624 |
| *Firmicutes Dorea* | 2.78219159 | 2.86516657 | 3.00437572 | 2.48857226 | 1.84044913 | 1.84848893 |
| *Firmicutes Enterococcus* | 0.30376181 | 0.04515419 | 0.01672187 | 0.00182565 | 0 | 0 |
| *Firmicutes Epulopiscium* | 0.00169163 | 0 | 0 | 0.02264535 | 0 | 0.01029969 |
| *Firmicutes Erysipelothrix* | 0 | 0 | 0 | 0 | 0.00052179 | 0 |
| *Firmicutes Erysipelotrichaceae* | 0.18816218 | 0.07867418 | 0.10341803 | 0.06215395 | 0.09054359 | 0.13340142 |
| *Firmicutes Eubacterium* | 0.43878141 | 0.41044378 | 0.31677699 | 0.22872045 | 0.08512371 | 0.3045325 |
| *Firmicutes Facklamia* | 0 | 0 | 0 | 0 | 0.00382649 | 0 |
| *Firmicutes Faecalibacterium* | 2.59766118 | 16.5227703 | 5.53451003 | 3.01696937 | 0.09859568 | 0.08631039 |
| *Firmicutes Filifactor* | 0 | 0 | 0 | 0 | 0 | 0 |
| *Firmicutes Gallicola* | 0 | 0 | 0.00069235 | 0 | 0 | 0 |
| *Firmicutes Gemellaceae* | 0 | 0 | 0 | 0 | 0 | 0 |
| *Firmicutes Helcococcus* | 0 | 0 | 0 | 0 | 0 | 0 |
| *Firmicutes Holdemania* | 0.02838386 | 0.02063345 | 0.0316427 | 0.02645838 | 0.00634702 | 0.00073578 |
| *Firmicutes Jeotgalicoccus* | 0 | 0 | 0 | 0 | 0.00574415 | 0 |
| *Firmicutes L7AE11* | 0 | 0 | 0.0189241 | 0 | 0 | 0 |
| *Firmicutes Lachnobacterium* | 0 | 0 | 0.00300016 | 0 | 0 | 0 |
| *Firmicutes Lachnospira* | 0.01122772 | 0.01361287 | 0.00651713 | 0.00068377 | 0.00524616 | 0 |
| *Firmicutes Lachnospiraceae* | 6.57649236 | 5.0811768 | 8.36786255 | 7.69957808 | 10.3061755 | 10.9469294 |
| *Firmicutes Lactobacillaceae* | 0.00323223 | 0 | 0 | 0 | 0 | 0 |
| *Firmicutes Lactobacillales* | 0.00072498 | 0 | 0 | 0 | 0.00092624 | 0 |
| *Firmicutes Lactobacillus* | 3.13949864 | 0.00566536 | 0.01924101 | 0.00153241 | 0.00224557 | 0.01599346 |
| *Firmicutes Lactococcus* | 0.00630538 | 0.00037976 | 0.00100293 | 0 | 0 | 0 |
| *Firmicutes Leuconostoc* | 0 | 0.0011422 | 0 | 0 | 0 | 0 |
| *Firmicutes Megamonas* | 0 | 0 | 0 | 0 | 0 | 0 |
| *Firmicutes Megasphaera* | 0.0204696 | 0.01320401 | 0.03202759 | 0.41973981 | 0.00851118 | 0.06812419 |
| *Firmicutes Mitsuokella* | 0 | 0.18571723 | 0.00420433 | 0.04576712 | 0 | 0.00381211 |
| *Firmicutes Mogibacteriaceae* | 0.09733466 | 0.05832047 | 0.67186238 | 0.84959786 | 1.91442686 | 1.80817252 |
| *Firmicutes Mogibacterium* | 0 | 0 | 0.00115391 | 0 | 0.00092624 | 0.00399994 |
| *Firmicutes Moryella* | 0 | 0 | 0.07915811 | 0 | 0 | 0 |
| *Firmicutes Oribacterium* | 0 | 0 | 0.00230782 | 0.00249987 | 0 | 0 |
| *Firmicutes Oscillospira* | 1.76859774 | 2.01425653 | 2.1103683 | 1.82502847 | 1.77278711 | 1.73414852 |
| *Firmicutes p75a5* | 0 | 0 | 0.02630911 | 0 | 0 | 0 |
| *Firmicutes Paenibacillus* | 0.00096665 | 0 | 0 | 0 | 0.00048119 | 0 |
| *Firmicutes Parvimonas* | 0 | 0 | 0 | 0 | 0 | 0 |
| *Firmicutes Pediococcus* | 0.01867615 | 0 | 0 | 0 | 0 | 0 |
| *Firmicutes Peptococcus* | 0 | 0 | 0 | 0 | 0 | 0 |
| *Firmicutes Peptostreptococcaceae* | 0.79722151 | 0.17638159 | 0.08464288 | 0.04716649 | 0.05841108 | 0.02904545 |
| *Firmicutes Peptostreptococcus* | 0 | 0 | 0 | 0 | 0 | 0.00179582 |
| *Firmicutes ph2* | 0 | 0 | 0 | 0 | 0 | 0 |
| *Firmicutes Phascolarctobacterium* | 0.15759377 | 0.27698829 | 0.27870464 | 1.12224027 | 0.71468189 | 0.62135397 |
| *Firmicutes Planococcaceae* | 0 | 0 | 0 | 0 | 0.09181291 | 0.44453527 |
| *Firmicutes Pseudobutyrivibrio* | 0 | 0 | 0.02123191 | 0 | 0 | 0 |
| *Firmicutes PseudoramibacterEubacterium* | 0.02277713 | 0.02558654 | 0.00387111 | 0.00773792 | 0.01490044 | 0.05193548 |
| *Firmicutes rc44* | 0.26706587 | 0.18575508 | 0.23756629 | 0.22134876 | 0.18536665 | 0.32752539 |
| *Firmicutes RFN20* | 0.04946449 | 0 | 0.02159307 | 0.09143746 | 0.08983463 | 0.12997924 |
| *Firmicutes Roseburia* | 0.02333411 | 0.02942365 | 0.03315304 | 0.09791175 | 0.02941462 | 0.09542885 |
| *Firmicutes Ruminococcaceae* | 28.9749286 | 14.6210493 | 22.2323556 | 10.3457717 | 18.7956389 | 15.6498695 |
| *Firmicutes Ruminococcus* | 2.88756724 | 2.90920738 | 2.85550918 | 4.15342415 | 4.86467916 | 4.06403803 |
| *Firmicutes Rummeliibacillus* | 0 | 0 | 0 | 0.00050334 | 0 | 0 |
| *Firmicutes Sarcina* | 0 | 0 | 0 | 0 | 0 | 0 |
| *Firmicutes Schwartzia* | 0 | 0 | 0.01015439 | 0 | 0 | 0 |
| *Firmicutes Selenomonas* | 0 | 0.00664576 | 0.01378006 | 0.011053 | 0.00486863 | 0 |
| *Firmicutes Sharpea* | 0.00619697 | 0.00376898 | 0.28096097 | 2.47598852 | 0.09849927 | 0.36088776 |
| *Firmicutes Shuttleworthia* | 0 | 0 | 0.14562323 | 0 | 0 | 0 |
| *Firmicutes SMB53* | 0.03289526 | 1.73393084 | 0.00040117 | 0 | 0 | 0 |
| *Firmicutes Solibacillus* | 0 | 0 | 0 | 0 | 0 | 0 |
| *Firmicutes Sporosarcina* | 0 | 0 | 0.00064082 | 0 | 0 | 0 |
| *Firmicutes Staphylococcus* | 0.00110271 | 0 | 0.00212019 | 0 | 0 | 0 |
| *Firmicutes Streptococcaceae* | 0 | 0 | 0.00081245 | 0 | 0 | 0 |
| *Firmicutes Streptococcus* | 0.34940336 | 0.12097942 | 0.03786236 | 0.00810084 | 0.00429954 | 0.02344561 |
| *Firmicutes Succiniclasticum* | 0.00057395 | 0 | 0.32982557 | 0 | 0 | 0.00084714 |
| *Firmicutes Tissierellaceae* | 0 | 0 | 0 | 0 | 0 | 0 |
| *Firmicutes Trichococcus* | 0 | 0 | 0 | 0 | 0.00191324 | 0 |
| *Firmicutes Turicibacter* | 0 | 0 | 0 | 0 | 0.00208517 | 0.00151757 |
| *Firmicutes Ureibacillus* | 0 | 0 | 0 | 0 | 0 | 0 |
| *Firmicutes Vagococcus* | 0 | 0 | 0 | 0 | 0 | 0 |
| *Firmicutes Veillonella* | 0 | 0 | 0 | 0 | 0 | 0 |
| *Firmicutes Veillonellaceae* | 0.01092869 | 0 | 0.06335506 | 0.01237284 | 0.15054882 | 0.10017742 |
| *Firmicutes Weissella* | 0 | 0 | 0.00115391 | 0 | 0 | 0 |
| *Fusobacteria Fusobacteriales* | 0 | 0 | 0 | 0 | 0 | 0 |
| *Fusobacteria Fusobacterium* | 0.00120831 | 0.00182018 | 0 | 0.00054096 | 0.00224684 | 0 |
| *Fusobacteria Leptotrichiaceae* | 0 | 0 | 0 | 0 | 0 | 0 |
| *Proteobacteria* | 0 | 0 | 0.00069235 | 0 | 0 | 0 |
| *Proteobacteria Achromobacter* | 0 | 0 | 0 | 0 | 0 | 0 |
| *Proteobacteria Acinetobacter* | 0 | 0 | 0 | 0 | 0 | 0 |
| *Proteobacteria Actinobacillus* | 0 | 0 | 0 | 0 | 0 | 0 |
| *Proteobacteria Agrobacterium* | 0 | 0 | 0.00200341 | 0.00256721 | 0.00115989 | 0 |
| *Proteobacteria Alcaligenaceae* | 0.0011948 | 0.00131494 | 0.00860714 | 0.00232496 | 0.0057255 | 0.00372232 |
| *Proteobacteria Alphaproteobacteria* | 0.01720504 | 0.0012554 | 0.00907733 | 0.00416577 | 0 | 0 |
| *Proteobacteria Amaricoccus* | 0 | 0 | 0.00137855 | 0 | 0 | 0 |
| *Proteobacteria Arcobacter* | 0 | 0 | 0.00046156 | 0 | 0 | 0 |
| *Proteobacteria Balneimonas* | 0 | 0 | 0 | 0 | 0 | 0 |
| *Proteobacteria Beijerinckiaceae* | 0 | 0 | 0.00140159 | 0 | 0 | 0 |
| *Proteobacteria Bilophila* | 0.00112158 | 0.0052819 | 0 | 0 | 0 | 0 |
| *Proteobacteria Bradyrhizobiaceae* | 0 | 0 | 0 | 0 | 0 | 0.00132899 |
| *Proteobacteria Brucellaceae* | 0 | 0 | 0 | 0 | 0 | 0 |
| *Proteobacteria Campylobacter* | 0 | 0 | 0 | 0 | 0 | 0 |
| *Proteobacteria Comamonas* | 0.05251313 | 0.0021058 | 0 | 0 | 0 | 0 |
| *Proteobacteria Desulfovibrio* | 0.00048332 | 0.00353056 | 0.00230782 | 0 | 0.00509702 | 0.00668193 |
| *Proteobacteria Desulfovibrionaceae* | 0 | 0 | 0 | 0 | 0 | 0 |
| *Proteobacteria Devosia* | 0.00217541 | 0.00153057 | 0.00162972 | 0.00036064 | 0 | 0 |
| *Proteobacteria Enterobacteriaceae* | 0.31616619 | 0.04586803 | 0.01511478 | 0.00054096 | 0.01554453 | 0.01972855 |
| *Proteobacteria Erythrobacteraceae* | 0 | 0 | 0 | 0 | 0 | 0 |
| *Proteobacteria Escherichia* | 0.02754159 | 0.01365726 | 0.00827201 | 0 | 0.01067214 | 0.00564822 |
| *Proteobacteria Gallibacterium* | 0.03176916 | 0.00056964 | 0 | 0 | 0 | 0 |
| *Proteobacteria GMD14H09* | 0 | 0 | 0 | 0 | 0 | 0 |
| *Proteobacteria Hylemonella* | 0 | 0 | 0.00069235 | 0 | 0 | 0 |
| *Proteobacteria Klebsiella* | 0 | 0 | 0 | 0 | 0 | 0 |
| *Proteobacteria Legionellaceae* | 0.00059081 | 0 | 0 | 0 | 0 | 0 |
| *Proteobacteria mitochondria* | 0 | 0 | 0.00184625 | 0 | 0 | 0 |
| *Proteobacteria Mycoplana* | 0 | 0 | 0 | 0 | 0 | 0 |
| *Proteobacteria Neisseriaceae* | 0 | 0 | 0 | 0 | 0 | 0 |
| *Proteobacteria Ochrobactrum* | 0 | 0 | 0 | 0 | 0 | 0 |
| *Proteobacteria Oligella* | 0 | 0 | 0 | 0 | 0.00121752 | 0 |
| *Proteobacteria Oxalobacter* | 0 | 0.00134091 | 0.00040622 | 0.00067909 | 0.00250847 | 0 |
| *Proteobacteria Paracoccus* | 0 | 0 | 0 | 0 | 0 | 0 |
| *Proteobacteria Pasteurellaceae* | 0 | 0.00089738 | 0 | 0 | 0 | 0 |
| *Proteobacteria Phyllobacteriaceae* | 0 | 0 | 0.00058563 | 0.00102161 | 0 | 0 |
| *Proteobacteria Proteus* | 0 | 0 | 0 | 0 | 0 | 0 |
| *Proteobacteria Providencia* | 0 | 0 | 0 | 0 | 0 | 0 |
| *Proteobacteria RF32* | 0.01286282 | 0 | 0.02794312 | 0.60750052 | 0.71876071 | 0.79106387 |
| *Proteobacteria Rhizobiaceae* | 0.00038584 | 0.00107685 | 0 | 0 | 0 | 0 |
| *Proteobacteria Rhizobiales* | 0 | 0.00156057 | 0.00056479 | 0.00284359 | 0 | 0 |
| *Proteobacteria Rhodobacter* | 0 | 0 | 0 | 0 | 0 | 0 |
| *Proteobacteria Rhodoplanes* | 0 | 0 | 0 | 0 | 0.00038721 | 0 |
| *Proteobacteria Rickettsiales* | 0 | 0 | 0 | 0 | 0 | 0 |
| *Proteobacteria Ruminobacter* | 0.00286751 | 0 | 0 | 0 | 0 | 0.01837772 |
| *Proteobacteria Sphingobium* | 0 | 0 | 0 | 0 | 0 | 0 |
| *Proteobacteria Sphingomonadales* | 0 | 0 | 0 | 0 | 0 | 0 |
| *Proteobacteria Succinivibrio* | 0.0267634 | 0.03758616 | 0.08997962 | 0.28483853 | 0.05237452 | 0.1658751 |
| *Proteobacteria Succinivibrionaceae* | 0 | 0 | 0.02077035 | 0 | 0.00044937 | 0 |
| *Proteobacteria Sutterella* | 0.41456595 | 0.15209118 | 0.44384571 | 0.79725005 | 1.04065748 | 0.81128209 |
| *Spirochaetes Sphaerochaeta* | 0 | 0 | 0.00092313 | 0 | 0 | 0 |
| *Spirochaetes Treponema* | 0 | 0 | 0.01546237 | 0.40034033 | 4.04143003 | 6.17355635 |
| *SR1* | 0 | 0 | 0.00230782 | 0 | 0 | 0 |
| *Synergistetes Synergistes* | 0 | 0 | 0 | 0 | 0 | 0 |
| *Synergistetes TG5* | 0 | 0 | 0 | 0 | 0 | 0 |
| *Tenericutes* | 0 | 0 | 0 | 0 | 0 | 0.0012707 |
| *Tenericutes Anaeroplasma* | 0 | 0 | 0.00276938 | 0 | 0.11546063 | 0.20577477 |
| *Tenericutes Anaeroplasmataceae* | 0 | 0 | 0 | 0.03467836 | 0.06015659 | 0.06375918 |
| *Tenericutes ML615J28* | 0.00477918 | 0 | 0 | 0.0094905 | 0 | 0 |
| *Tenericutes Mollicutes* | 0 | 0 | 0 | 0.01220207 | 0.01483901 | 0.02078038 |
| *Tenericutes Mycoplasmataceae* | 0 | 0 | 0.00069235 | 0 | 0 | 0 |
| *Tenericutes RF39* | 0.02971152 | 0.06938161 | 0.11706534 | 0.21854887 | 0.2473044 | 0.62301602 |
| *TM7* | 0 | 0 | 0 | 0 | 0 | 0 |
| *TM7 EW055* | 0 | 0 | 0 | 0 | 0 | 0 |
| *TM7 F16* | 0 | 0 | 0.03438647 | 0 | 0 | 0 |
| *Verrucomicrobia Akkermansia* | 0.0287035 | 0.0462633 | 0.07225874 | 0.00410771 | 0.00064159 | 0.01195544 |
| *Verrucomicrobia Cerasicoccaceae* | 0 | 0 | 0 | 0 | 0.0225583 | 0 |
| *WPS2* | 0 | 0 | 0.00438485 | 0 | 0 | 0 |
| *Tenericutes Mycoplasmataceae* | 0 | 0 | 0.00069235 | 0 | 0 | 0 |
