# Supplementary figures and images for: Temporal changes in the fecal bacterial community in Holstein dairy calves from birth through the transition to a solid diet
Source: PLoS One. 2020 Sep 8;15(9):e0238882. doi: 10.1371/journal.pone.0238882 (PMC7478546; doi:10.1371/journal.pone.0238882)

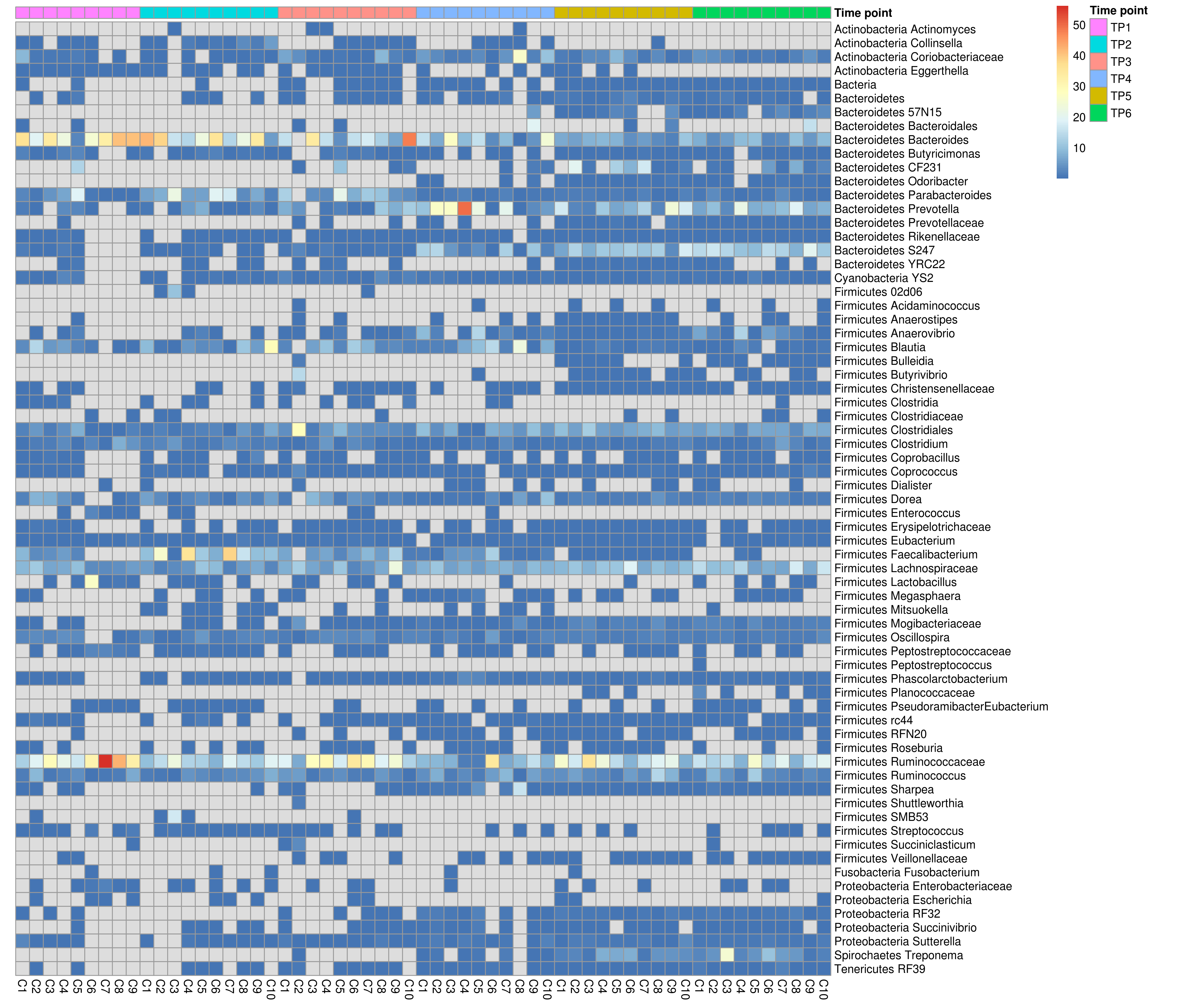

Supplement: S1 Fig — Heat map showing the relative abundance of genera in each of the 10 calves (C1-C10, bottom axis) sampled at each of the 6 study timepoints (TP, top axis). Relative abundance (%) of each genus is indicated by color (color scale given in upper right-hand corner). The symbol # beside genus name indicates unclassified genus from the family, order, class, phylum, or kingdom given. Grey squares indicate absence of a genus in that sample. (TIFF) [file pone.0238882.s007.tiff]
